# Ab initio simulation of the absorption spectrum of Ruby and the role of vibronic coupling

**DOI:** 10.1007/s00894-026-06985-0

**Published:** 2026-10-14

**Authors:** Leon D. Starke, Amanda D. Torres, Alexandre B. Rocha

**Affiliations:** Chemistry Institute, Federal University of Rio de Janeiro, Avenida Athos da Silveira Ramos, 149, Centro de Tecnologia, Rio de Janeiro, 21941-909 Rio de Janeiro Brazil

**Keywords:** Ab-initio methods, Solid-state, Vibronic-coupling, Spectroscopy

## Abstract

**Context:**

Ruby is a material commonly found in Nature. Its red color is due to chromium impurity in the lattice of α-alumina. It has several technological applications, such as lasers, thermometers, and radiation dosimeters. The study of its absorption spectrum is relevant in this context. On the other hand, vibronic coupling in solids is an important phenomenon since it can change the features of the absorption spectrum, especially when involving transition metal *d-d* electronic transitions in a quasi-octahedral environment, as in the case considered here. This work provides insight into the role of the vibronic coupling mechanism in the profile of the absorption spectrum of Ruby in the visible region.

**Methods:**

The spectrum of Ruby, i.e., α-alumina doped with chromium, is calculated within the linear response regime applied to a PBE DFT initial calculation. Intensities are taken as the imaginary part of the frequency-dependent dielectric function, obtained by the Green-Kubo formula. The model is composed of an α-alumina supercell in which an aluminum atom is replaced by a chromium atom. Periodic boundary conditions were applied throughout. The vertical spectrum at the equilibrium geometry was first reported. Then, vibronic coupling effects were evaluated by carrying out distortions of the geometry along the normal modes related to the motions of chromium and its first neighbors. In addition, the effect of distortions involving combined modes was also assessed. In the last case, the modes are combined in pairs, generating hundreds of possibilities, all of which were carefully calculated. Modes (pure and combined) that change the absorption profile are clearly identified, and it is shown that they were able to change both position and intensity of the absorption peak in the visible region.

**Supplementary Information:**

The online version contains supplementary material available at https://doi.org/10.1007/s00894-026-06985-0.

## Introduction

The most stable form of Al$_2$O$_3$ is its α phase [1]. When doped with chromium, it forms Ruby, which is an important material with several technological applications. One of the first and most important applications is the fabrication of solid-state lasers [2]. This application is only possible due to the existence of the R lines, which are transitions forbidden by the spin selection rule [3]. Ruby can also be used as a thermometer [4] and a radiation dosimeter [5]. It is resistant to chemical attack and presents good thermal conductivity and thermal resistance [6]. Alone, α-alumina has no color, but when a transition metal atom, like chromium, isomorphically substitutes an aluminum atom as an impurity in the lattice, the resulting material absorbs in the visible spectra region [7]. The ion $\text {Cr}^{3+}$ has a *d*$^3$ configuration. Following the *d*$^3$ Tanabe-Sugano diagram, it would be expected to have five allowed and two spin-forbidden transitions [8]. Nevertheless, only two absorptions are observed in the visible window, attributed to transitions among quartets in this oversimplified description [9].

It is possible to simulate this absorption spectrum using embedded methods, in which a cluster of atoms is constructed, and a quantum mechanical calculation for those atoms is performed. The other atoms surrounding the cluster are represented by point charges that mimic the potential due to the rest of the lattice. Ogasawara et al. [10] used a cluster model within DFT coupled with configuration interaction to describe Ruby. A many-electron Hamiltonian was constructed, and the authors used three distinct methods for the calculation of the generated matrix elements. A correlation correction factor was considered to further approximate the correlation effects. Oscillator strengths were calculated. All methods yielded two bands in the absorption spectra, but different peak positions depending on which approach was used.

Watanabe et al. [11] also used an embedded cluster in the formalism of DFT to generate molecular orbitals and then performed a configuration interaction to obtain multiplet energies. Likely to Ogasawara et al. [10], a configuration-dependent correlation correction was used. The authors successfully obtained two bands in the visible spectra.

Novita et al. carried out [12] a similar idea using an embedded cluster for Ruby. The absorption spectra for Ruby were simulated by considering a distinct electronic configuration, followed by the configuration interaction method, which yielded three bands with two in the visible region.

Andreici et al. [13] also used an embedded method for Ruby, considering explicitly only the chromium atom and its oxygen first neighbors. But now, the properties of the system were calculated using CASSCF, and excited-state energies were improved with electron correlation methods. The two absorption peaks were observed in this simulation, and since an embedded method was used, it shows how the main features in absorption spectra occur within the CrO$^{-9}_6$ cluster.

Instead of an embedded cluster, another possible approach to the problem is to use periodic DFT with plane waves basis set, as done by Na-Phattalung et al. [14], who have calculated the energy of impurity formation with the HSE hybrid functional. They have also indirectly estimated the gap by changing the number of electrons associated with the impurity. They have not calculated the absorption spectrum explicitly, though. A DFT-LDA+U [15] approach was successful in simulating the band gap of the material and showed how the band gap diminished when chromium is introduced in α-alumina.

Saito et al. [16] also used the periodic approach. Initially, they constructed a supercell to optimize the structure of Cr-doped alumina. Then, a cluster of atoms was selected. This cluster has a charge that was neutralized by a background of compensating charges to perform a GW calculation on the cluster in order to construct an effective Hamiltonian for the *d* electrons in Ruby. This was done since the GW in a supercell is computationally quite demanding. Hence, they permit the variation of six different parameters in that effective Hamiltonian. This method permits the extraction of pertinent crystal field parameters and multiplets. In that work, neither the absorption nor the emission spectra were explicitly calculated.

On the experimental side, Drdlikova et al. [9] recorded the absorption spectra of Ruby and found one peak at 2.22 eV and the second one at 3.06 eV, both attributed to the chromium impurity *d-d* transitions in the lattice. Hiroshi et al. [6] got a similar result from absorption spectra with peaks absorbing at 2.25 eV and 3.10 eV. They observe that the intensity of the peaks is a function of Cr$^{3+}$ concentration in the alumina, with an increase of intensity up to a certain limit [9, 15], with the intensity of emission spectra following the same trend. Abd Halim et al. [15] showed that the maximum of intensity absorption in Ruby occurs with 0.8 wt% of Cr.

The absorption in the visible region by Ruby is a consequence of the *d-d* transitions at the dopant atom. Consequently, distortions of the geometry around the chromium atom should change the absorption spectra. Therefore, this seems to be a good system to simulate vibronic coupling since we can probe the answer to the distortions in the system by directly looking at the absorption spectra.

For an electronic transition to be allowed, the direct product of the irreducible representations of the ground and excited state must contain the totally symmetric term [17]. However, even if a pure electronic transition is forbidden by symmetry, when we introduce a dependence on the vibrational wavefunction, the transition can now occur. Hence, the transition is said to be vibronically allowed, which happens, for example, with *d-d* transitions in molecules with an inversion center [17]. It should be emphasized, though, that even when *d-d* transitions take place in a non-octahedral local symmetry, and consequently the transition is not strictly forbidden, the vibronic interaction is important in changing the shape and intensity of the peaks. That is precisely the case here.

As mentioned before, the calculation of the absorption spectrum of impurity levels is commonly done by making use of embedded cluster models [10–12]. Nevertheless, in this work we use a periodic impurity system with a large unit cell, an approach much less exploited to date [14]. As far as we are concerned, this is the first time the Ruby absorption spectrum has been obtained at this level. Furthermore, not only will the calculated spectrum at the equilibrium geometry be shown, but we will also analyze the effect of the vibronic coupling in the electronic absorption spectrum in the visible region. The treatment will be mostly qualitative, but it was inspired by our previous work on a quantitative method known as direct vibronic coupling (DVC) [18, 19]. Instead of calculating the optical oscillator strength directly from the transition moment as in the previous work, the intensity would be extracted from the imaginary part of the frequency-dependent dielectric function. This function tells us how a material responds to an external electric field and, in the case of an insulator, like α-alumina, how the material polarizes. Its imaginary part is associated with the dissipation of energy by the dielectric and therefore with the absorption spectra. In this sense, this quantity is related to the density of allowed optical transitions and consequently proportional to the probability of electronic transitions. The imaginary dielectric function can be used to map the vibronic coupling in systems like Ruby by calculating it along the normal coordinates of vibration, in which the vibronic coupling phenomenon can be evidenced. This was done both in pure normal mode displacements and in combined modes, in order to verify the contribution of cross-terms.

## Computational details

All calculations were done in the software VASP[20] at the DFT level with the correlation-exchange functional PBE [21] and plane waves basis set [21]. Brillouin zone summations were done in a Monkhorst–Pack 4 X 4 X 4 k-point sampling for the supercell. The cutoff energy was 400 eV, and the energy convergence threshold was $10^{-6}$ eV. For the Projected Density of States (PDOS) calculation, an 800 eV cutoff energy was used. Core electrons were treated within the Projected Augmented Waves (PAW) approximation [22]. The initial geometry for α-alumina was extracted from the Crystallography Open Database [23]. The lattice parameters of the cell used were: $a = b = 4.76\,\mathring{A}, \quad c = 12.99\,\mathring{A}, \quad \alpha = \beta = 90^\circ ,\; \gamma = 120^\circ$.

From the α-alumina primitive cell, a supercell containing 30 atoms was generated, and one of the aluminum atoms was substituted by chromium in order to generate a model system to represent Ruby. The final cell has 11 aluminum, 1 chromium, and 18 oxygen atoms. The geometry was optimized by using the conjugate gradient algorithm, and the optimization allowed the atoms to change position while maintaining the shape and volume of the unit cell.

Then, a partial phonon calculation was done, in which only the chromium atom and its six nearest oxygen atoms were allowed to move. We have obtained 21 real frequencies, as expected.

For the electronic absorption spectra, the imaginary part of the frequency-dependent dielectric function was calculated within the linear response regime by using the Green-Kubo formula, which implies a summation over empty states. The procedure also includes broadening of the dielectric functions by Gaussian and Lorentzian smearing functions [24]. This method is a compromise between accuracy and computational cost, since hundreds of calculations were performed, as explained in what follows.

Additionally, by calculating the dielectric function on distorted geometries along the normal coordinates of vibration, the vibronic coupling phenomenon can be evidenced. The core idea is to systematically distort the system and analyze the effects on the spectra.

To generate the geometries used, we distort the atoms of the system in the directions of the displacement vectors of the calculated phonons in different extensions and calculate the imaginary part of the dielectric function for each deformation. So, for all 21 normal modes, we generated 63 new geometries by changing atom position in 10, 30, and 50% of the maximum displacement of each mode. We also combined those modes in pairs, considering distortions in the positive and negative directions of the displacement vectors. So, we generated more than 600 new geometries with a combination of modes with a distortion of 25% for each mode. Finally, the average spectra are obtained for both pure and mixed distortions. We generated combinations of both positive and negative directions of the displacement vectors, which will be labeled pp and nn modes, and combinations with one along the positive and the other in the negative direction of the phonon, which will be called pn and np modes.

The above procedure is largely inspired by the Direct Vibronic Coupling Method (DVC) we have used before [18].

## Results

**Fig. 1:**
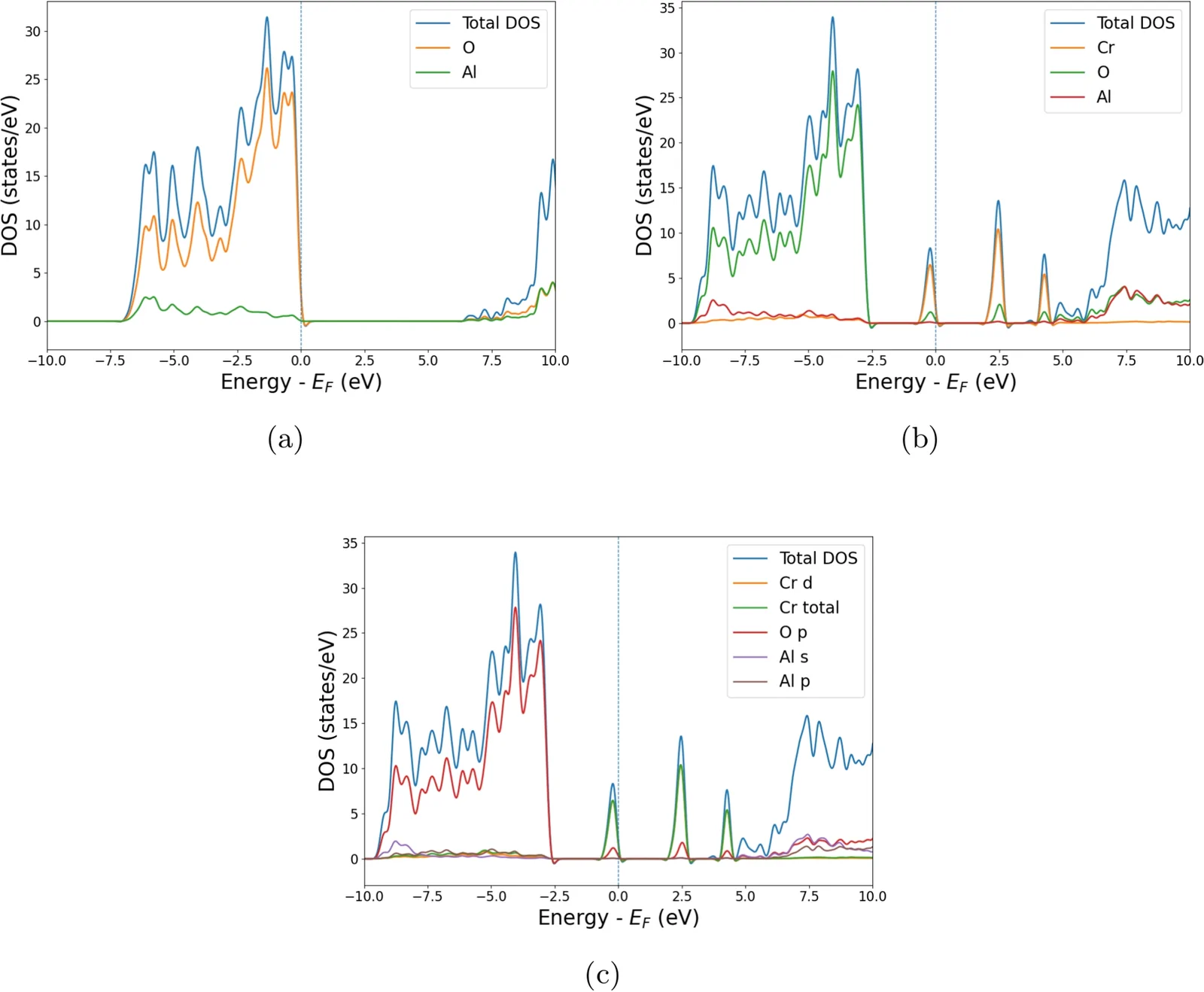
Projected density of states: **a** PDOS for pure α-Al$_2$O$_3$, **b** element-resolved PDOS for Cr-doped α-Al$_2$O$_3$, and **c** orbital-resolved PDOS for Cr-doped α-Al$_2$O$_3$

The first important result is the comparison of the PDOS of pure α-alumina (Fig. 1a) and of α-alumina with chromium (Fig. 1b. Substituting $\text {Al}^{3+}$ by $\text {Cr}^{3+}$ introduces a defect center whose electronic structure differs qualitatively from that of the host lattice. Not only is the band gap diminished, but the impurity also gives rise to narrow localized electronic states composed mainly of 3*d* chromium orbitals, as can be observed in Fig. 1c. These states are responsible for the optical transitions in the visible spectra of Ruby [25]. The calculated PDOS is in good agreement with those reported in the literature by Wallin et al. [26]. It is worth observing that the shift of the projected density of states for oxygen and aluminum when chromium is introduced occurs because the states are plotted relative to the Fermi energy.

**Fig. 2:**
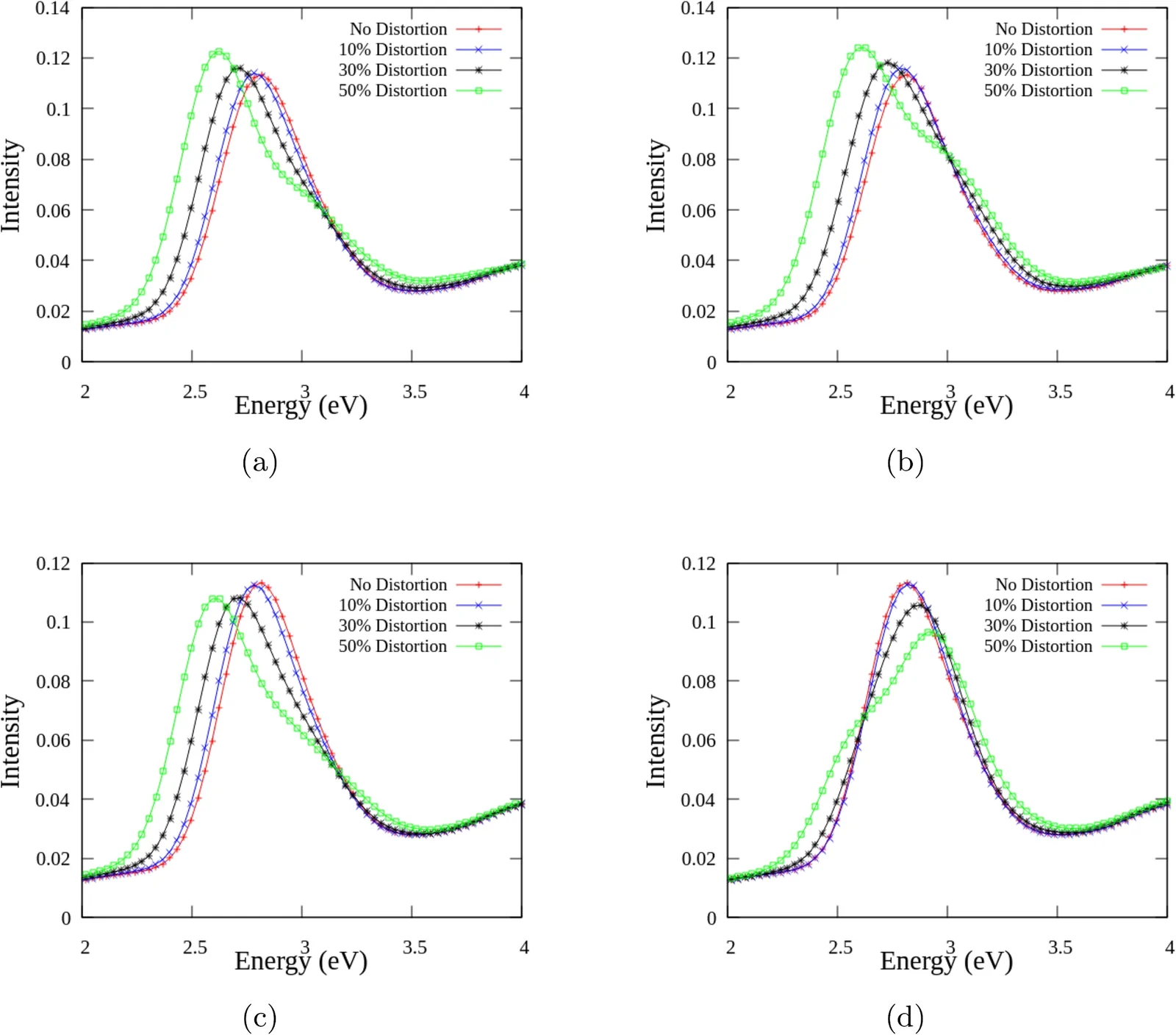
Imaginary part of the frequency-dependent dielectric function of the spectra in which a second peak could be observed: Spectra generated from pure modes for frequencies **a** 718.93 cm$^{-1}$, **b** 718.36 cm$^{-1}$, **c** 657.88 cm$^{-1}$, and **d** 657.64 cm$^{-1}$

Since the doped α-alumina does not have a local octahedral symmetry [27], the *d-d* transitions from chromium are not forbidden by the Laporte rule [28]. In this way, they should absorb in the blue region of visible spectra, giving the characteristic red color of Ruby [29]. Although these electronic *d-d* transitions are allowed, they can change when considering vibronic interactions. The PDOS graph shows electronic states, mainly composed of Cr *d* states close to the band gap, which strongly correlate with the absorption in the visible spectra to Cr-centered transitions [30]. Hence, we restricted our analysis to the chromium atom and its six oxygen neighbors. A similar problem of quasi-forbidden transitions was analyzed in the molecular case for methyl formate [31].

We present the calculated spectrum of Ruby initially at the vertical approximation, i.e., obtained at the equilibrium geometry. Also, we investigate how the spectrum of the system responds to distortions along normal modes and identify those that contribute more to the vibronic coupling phenomenon. The analysis is done by plotting the imaginary part of the frequency-dependent dielectric function as a function of the absorption energy. Consequently, we can access the electron–phonon interaction through the optical response. Figure 2 shows the imaginary part of the frequency-dependent dielectric function calculated for some selected normal modes, compared with the equilibrium geometry (no distortion). Heiba et al. [33] used the PBE functional to calculate the electronic properties of ruby, more specifically the real and imaginary parts of the dielectric function with success. Ahmed et al. [34] computed the same properties already mentioned also with the PBE functional. Those results show that PBE is a suitable choice for the system that is being studied. Some points deserve attention. First, the profile of the imaginary part of the dielectric function, obtained in the equilibrium geometry, does not present two peaks. This is due to limitations of the calculation method. As we have already mentioned, the procedure involves a broadening scheme by Gaussians and Lorentzians. The resulting absorption bands are consequently widened and merged into a single band, with a maximum around 2.8 eV, in between the experimental peaks at 2.2 and 3.1 eV. However, as seen on spectrum profiles shown in Fig. 2, when distortion is applied, these two bands separate in energy, with a second one appearing as a shoulder on the right-hand side of the primary band. These results agree, in general, with the experimental spectra done by Esposti et al. [3], in which two peaks are clearly observed in the range 2 and 4 eV. This phenomenon was not observed for all modes. The modes that did not exhibit it were also analyzed, although they are not shown in the figure. It is important to point out that this work aims to understand the extension of vibronic coupling in ruby and not to reproduce exactly the experimental spectra. The relative variation of the bands in the visible region was analyzed using the same method for undistorted and distorted structures, so the shift in the peak centered in 2.8 eV can be used as a semi-quantitative parameter on the sensitivity of electronic properties with respect to structural perturbations. A DFT+U calculation was done (all spectra are available in Supplementary Information, Fig. S1), and although the split of bands appears, some nonphysical results also emerge, such as a significant part of the spectra has negative intensities and, also, the intensities of the two bands in the visible region are way too low and therefore close to the method error.

**Fig. 3:**
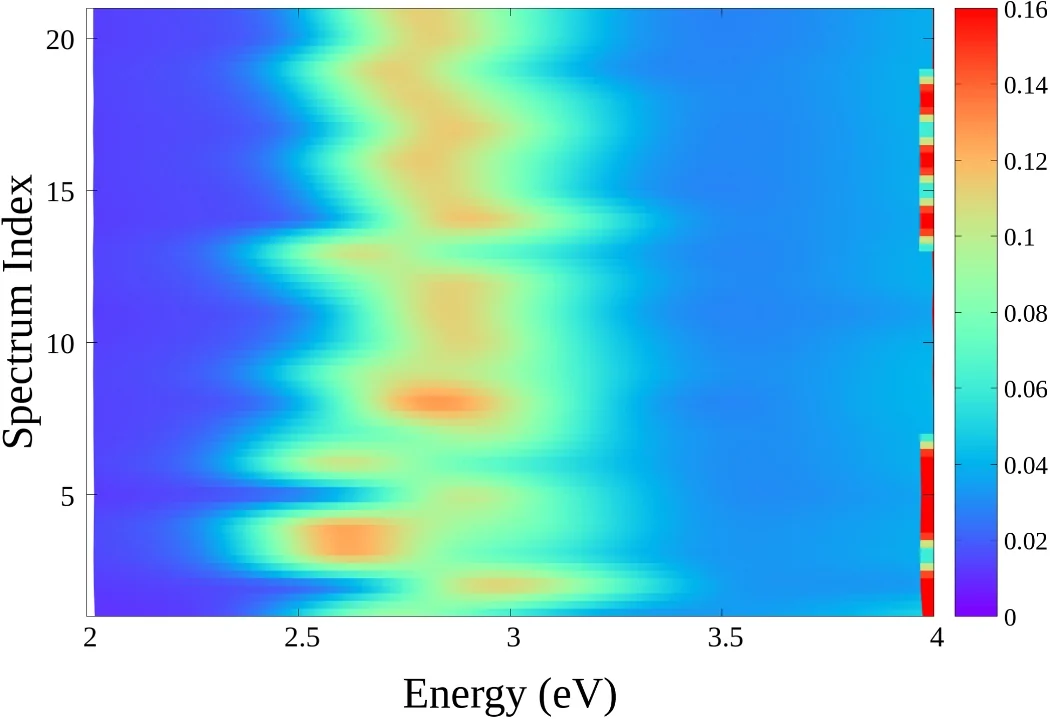
Heat map for pure modes with 50% distortion. The *y*-axis is the index of spectra, with the colors indicating the intensity of absorption in the respective *x* value

**Fig. 4:**
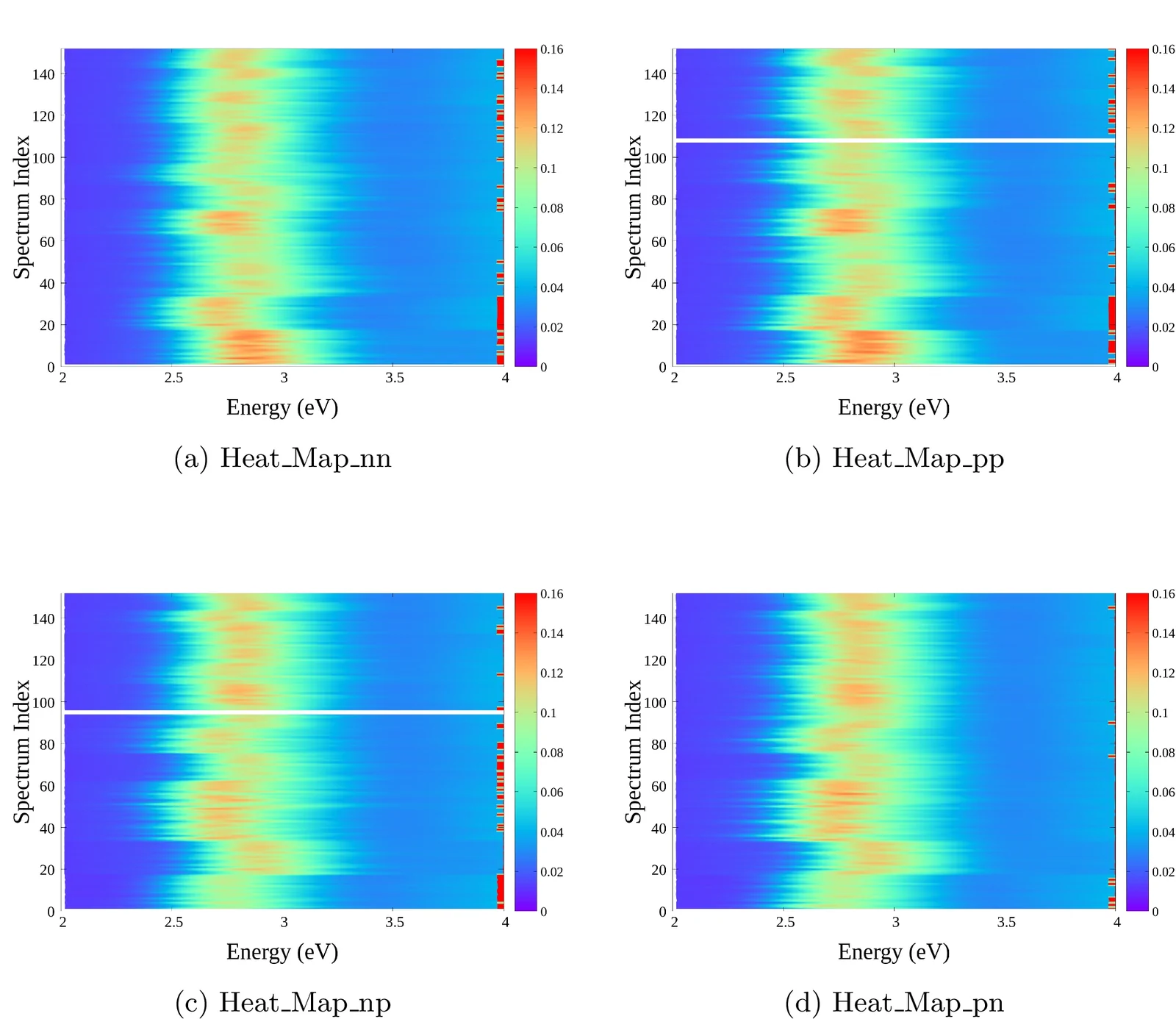
Heat maps of crossed terms: the *y*-axis is the index of spectra, with the colors indicating the intensity of absorption in the respective *x* value. The heat maps were done for all combinations of distortions: nn, pp, np, and pn. The blank lines in both np and pn spectra exist because it was not possible to calculate the imaginary of the frequency-dependent dielectric function for two specific distortions

**Fig. 5:**
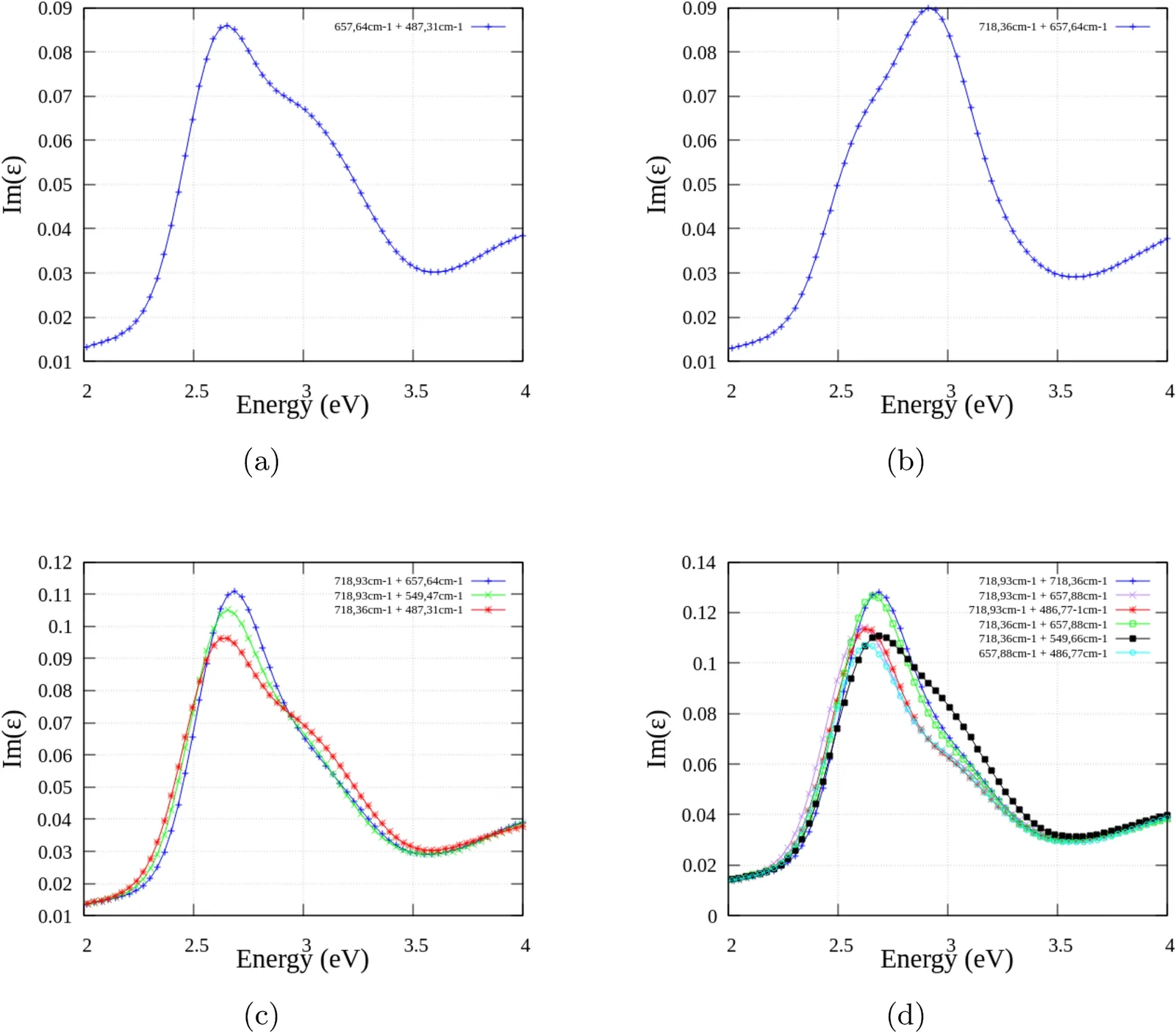
Imaginary part of the frequency-dependent dielectric function of the spectra where a second peak could be observed within combined modes: **a** Spectra generated from nn combined distortions, **b** Spectra generated from np combined distortions, **c** Spectra generated from pn combined distortions, **d** Spectra generated from pp combined distortions

**Fig. 6:**
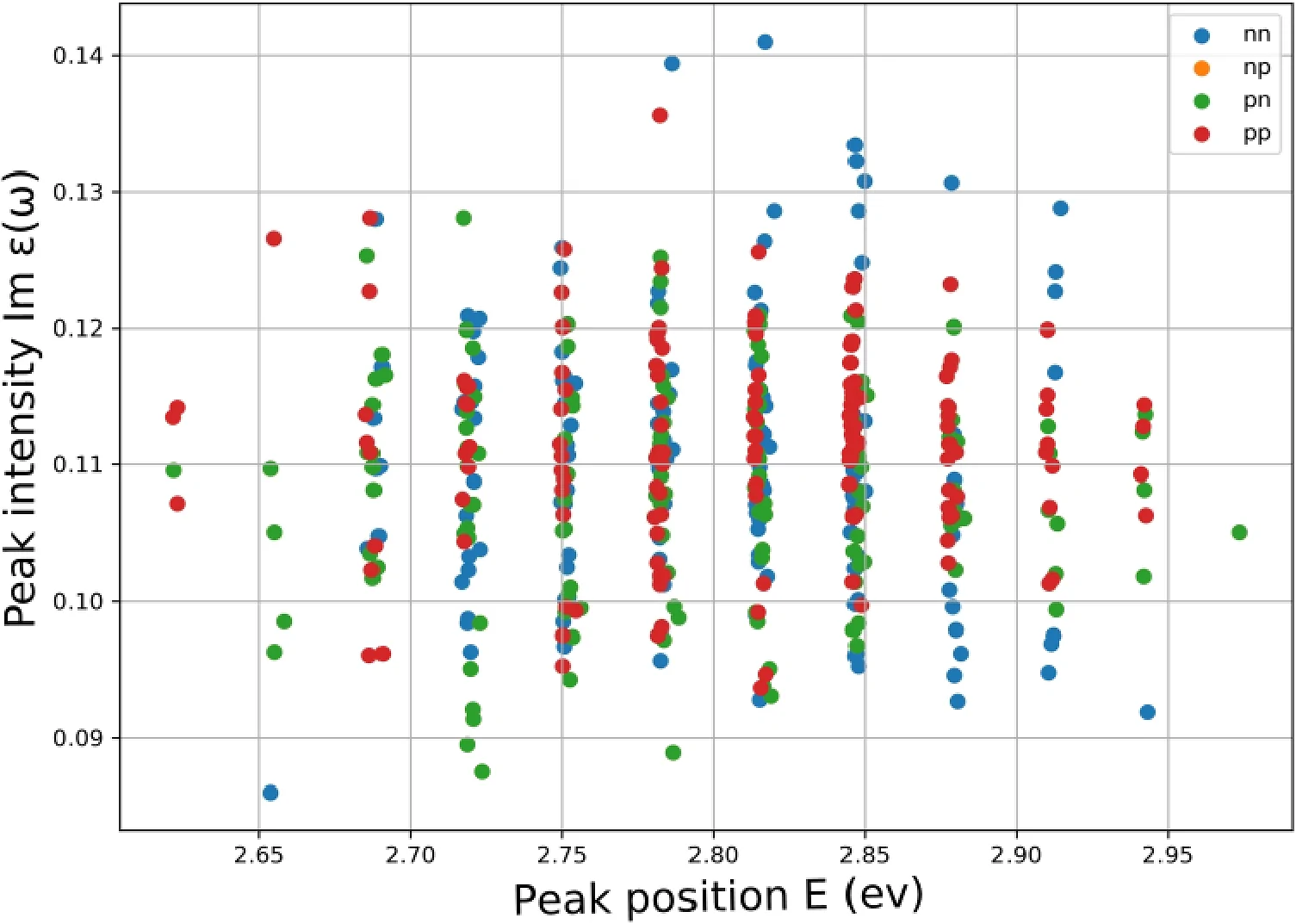
Peak position and intensities of all crossed modes

The appearance of those features is related to symmetry breaking of the CrO$^{-9}_6$ structure. The local symmetry of chromium in Ruby is C$_3$ [27], and therefore the analysis of the optical transition using d$_3$ Tanabe-Sugano diagram is merely qualitative since there is no octahedral symmetry. Even so, the literature attributes the peaks in visible spectra to the $^4A_2$ → $^4 T_1$ e $^4A_2$ → $^4T_2$ transitions [3].

It is possible to classify the normal modes that were obtained from our calculations in several categories: symmetric and antisymmetric stretching, scissoring (bending), rocking, twisting, and wagging. This is a qualitative analysis, and more than one category can fit for the same normal mode. A table with all classified modes can be found in the Supplementary Information as Table S4. It is observed that the modes which changed the most the dielectric function (778.36; 720.62; 661.32; 616.60; 549.66; 549.47; 430.94 cm$^{-1}$) and those that not only modified the absorption spectra but made another peak appear as a shoulder (718.93; 718.36; 657.88; 657.64 e 486.77 cm$^{-1}$) were all composed of scissoring or stretching modes. On the other hand, those that barely changed the spectra (503.80; 487.31; 422.50; 422.43; 365.43; 364.81; 269.50; 268.88 cm$^{-1}$) are composed mainly of rocking, wagging, and twisting. From our results, it is clear that scissoring and stretching modes are able to produce larger changes in the absorption spectra. The reason behind that is the fact that those modes alter both Cr-O bond length and O-Cr-O angles and therefore are able to break the symmetry of the system even further, activating vibronic interactions that lead to greater alterations in the dielectric function. The same cannot be said for rocking, wagging, and twisting mainly because they produce only small changes in the coordination environment.

As pointed out by Zaitsev [32], due to the small size of a substituted atom, such as chromium in α-alumina, vibronic coupling involving that defect is more effective with higher wavenumber modes, which is in agreement with what we have found here. It is important to note that larger distortions cause larger changes in the spectra. Even larger distortions (larger than 50%) were avoided since at distances far from the equilibrium geometry the vibrational function drops to zero [35].

All spectra distorted in an extension of 50% were used to construct the heat map shown in Fig. 3. This map will be used in a qualitative analysis of the dielectric function in what follows.

The analysis of the spectra resulting from combined distortions in pairs by 25% in each normal mode was also done. Since this procedure generated more than 600 spectra, heat maps were constructed to provide a more accessible overview of the results. One heat map was generated for each distortion type (nn, pp, np, and pn), and all four are presented in Fig. 4.

It is also evident that the four types of distortions are not equivalent. This is a consequence of the fact that different kinds of distortions break the symmetry of the system differently. The nn and np distortions generated peaks between 2 and 4 eV with higher intensities when compared with pn and pp. In all spectra, we can observe that some modes can change the position of the visible peak. From the heat map of the pure modes (Fig. 3), it is also possible to observe changes in both intensity and peak position with modifications within low-index spectra and higher wavenumbers, as it was expected [32]. Comparison of the heat maps: crossed modes, especially nn and np, generated higher-intensity spectra than pure modes. A very interesting thing to notice is that, overall, modes that provide a red shift in nn and np are blue-shifted in pp and pn. In the same way, those that provide a blue shift in nn and np are red-shifted in pp and pn. This led to the fact that the np and nn heat maps are just about the mirror images of the pn and pp heat maps.

It is possible to see that a few crossed modes generated a shoulder in the spectra or even a second peak. Therefore, even though distortions in crossed modes were done by the sum of contributions of only 25% from each normal mode, it is already possible to see symmetry breaking upon combination. In this sense, a larger distortion for crossed terms could generate even greater symmetry lifting. After plotting all spectra, thirteen of them were sorted from the hundreds because those showed a second peak or even a slight shoulder appearing on the graph, all shown in Fig. 5. The frequencies from the modes that contributed to the appearance of a second peak in crossed modes are the same ones that made another peak emerge when analyzing pure modes. In three combined modes, one of the frequencies assembled in the pair did not change the spectra alone when looking at pure modes. These frequencies are 487.31, 549.47, and 549.66 cm$^{-1}$. Although they cannot change the spectra alone, they were paired with another mode that effectively changed the spectra when solo. To sum up, the modes that made another peak emerge when they were alone still made the same when paired with others. This reinforces the idea that there are specific frequencies that couple with the electronic states and therefore change the absorption spectra, lifting the symmetry of the system.

**Table 1:** Statistical analysis of peak energy and intensity in the 2–4 eV window

	pn	np	pp	nn
Peak energy (eV)				
Mean	2.792620	2.792620	2.807399	2.799319
Std. deviation	0.071032	0.071032	0.069067	0.059820
Minimum	2.622000	2.622000	2.621700	2.653700
Maximum	2.973600	2.973600	2.942900	2.943400
Energy CV (%)	2.544	2.544	2.460	2.137
Peak intensity Im				
Mean	0.108514	0.108514	0.111482	0.110024
Std. deviation	0.007876	0.007876	0.007296	0.009846
Minimum	0.087500	0.087500	0.093700	0.086000
Maximum	0.128100	0.128100	0.135600	0.141000
Intensity CV (%)	7.258	7.258	6.545	8.949

As we can observe from both Fig. 6 and Table 1, we have a broad fluctuation in the position of the peak, roughly of 0.34 eV. Also, almost a 40% difference in peak intensity was observed, indicating that the distortions made had a great influence on the oscillator strength. It is possible to observe in Fig. 6 that the peak positions are gathered vertically with many corresponding intensities, which leads to the conclusion that different distortions result in close peak position energy.

Because the system was distorted using fixed amplitudes, it is expected that the data gathers in this way. On the other hand, if the points were equally spaced along all peak positions, it would be fair to assume that the dielectric function has an isotropic response to the applied distortions, which is clearly not the case. Instead, the persistent vertical clustering in narrow gaps suggests that the dielectric function responds in an anisotropic way to nuclear motion, with response dominated by certain distortions. The graph shows no apparent correlation between intensity and peak position. Hence, those quantities respond differently and independently to distortions in the system.

## Conclusions

In this work, we have presented a systematic analysis of the vibronic coupling effect in the absorption spectrum of α-Al$_2$O$_3$ doped with chromium as a model for Ruby. Initially, the projected density of states (PDOS) showed that the insertion of Cr$^{3+}$ generates narrow and localized states within the band gap.

Then, the spectrum was calculated from the imaginary part of the frequency-dependent dielectric function plotted as a function of the absorption energy. The method does not rely on the calculation of individual transitions, but generates the dielectric function as a continuum function of the transition energy. This tends to broaden the bands, diminishing the resolution of the spectra. While this is a clear disadvantage, the computational efficiency is advantageous, especially by considering the hundreds of calculations performed in order to evidence the vibronic coupling. The latter is revealed by distorting the geometry along pure and combined normal modes and analyzing the changes in the spectrum profile. This procedure made emerge the activating modes. Some of them changed both the intensity and the peak position. Also, some modes broke the symmetry of the system, making another peak visible as a shoulder. So, the broadening of the bands does not prevent the appearance of vibronic effects, since the profile was significantly changed in those cases.

Heat maps showed approximate mirroring between nn/np and pp/pn distortions, indicating partial symmetry-related effects. By analyzing the peak position versus intensity of all crossed modes, we see that the optical response occurs only for some specific distortions rather than being evenly distributed. Hence, vibronic coupling in the studied system is mode-selective. For combined modes, we observed a wide variation in both peak position and intensity, with those quantities responding independently. To sum up, we have performed an analysis that was able to track vibronic coupling by directly looking at the effect on the absorption spectra.

## Supplementary Information

Below is the link to the electronic supplementary material.

Supplementary file 1 (pdf 399 KB)

## Data Availability

No datasets were generated or analysed during the current study.

## References

[1] Li S, Wang P, Hu J, Yang G, Lu Y, Yuan Z, Zhang X, Liang S (2025) Synthesis and application study of -AlO with large specific surface area at high temperature by hydrothermal-assisted freeze-drying. Ceram Int 51:2163–2172. 10.1016/j.ceramint.2024.11.193

[2] Maiman TH (1960) Stimulated optical radiation in ruby. Nature 187:493–494. 10.1038/187493a0

[3] Degli Esposti C, Bizzocchi L (2007) Absorption and emission spectroscopy of a lasing material: ruby. J Chem Educ 84:1316. 10.1021/ed084p1316

[4] Grattan KTV, Selli RK, Palmer AW (1988) Ruby decay-time fluorescence thermometer in a fiber-optic configuration. Rev Sci Instrum 59:1328–1335. 10.1063/1.1140257

[5] Einbergs E, Zolotarjovs A, Bite I, Laganovska K, Auzins K, Smits K, Trinkler L (2019) Usability of Cr-doped alumina in dosimetry. Ceramics 2:525–535. 10.3390/ceramics2030040

[6] Murotani H, Mituda T, Wakaki M, Kondou Y (2000) Optical characteristics of AlO ceramics doped with Cr at high concentrations prepared by extrusion molding process. Jpn J Appl Phys 39:2748. 10.1143/JJAP.39.2748

[7] Zhu YL, Liu S, Zhang XK, Xiang Y (2018) Effects of Cr concentration on the crystallinity and optical properties of Cr-doped AlO powders by solid-state reaction method. IOP Conf Ser Mater Sci Eng 382:022037. 10.1088/1757-899X/382/2/022037

[8] Brik MG, Camardello SJ, Srivastava AM, Avram NM, Suchocki A (2016) Spin-forbidden transitions in the spectra of transition metal ions and nephelauxetic effect. ECS J Solid State Sci Technol 5:3067–3077. 10.1149/2.0091601jss

[9] Drdlikova K, Klement R, Drdlik D, Galusek D, Maca K (2020) Processing and properties of luminescent Cr-doped transparent alumina ceramics. J Eur Ceram Soc 40:2573–2580. 10.1016/j.jeurceramsoc.2019.11.010

[10] Ogasawara K, Ishii T, Tanaka I, Adachi H (2000) Calculation of multiplet structures of Cr and V in -AlO based on a hybrid method of density-functional theory and configuration interaction. Phys Rev B 61:143–161. 10.1103/PhysRevB.61.143

[11] Watanabe S, Sasaki T, Taniguchi R, Ishii T, Ogasawara K (2009) First-principles calculation of ground- and excited-state absorption spectra of ruby and alexandrite considering lattice relaxation. Phys Rev B 79:075109. 10.1103/PhysRevB.79.075109

[12] Novita M, Ogasawara K (2012) Comparative study of absorption spectra of V, Cr, and Mn in -AlO based on first-principles configuration-interaction calculations. J Phys Soc Jpn 81:104709. 10.1143/JPSJ.81.104709

[13] Andreici Eftimie EL, Avram NM (2020) Absorption spectra, ligand field parameters and g factors of Cr-doped -AlO laser crystal: ab initio calculations. Phys Scr 95:044005. 10.1088/1402-4896/ab60fc

[14] Na-Phattalung S, Limpijumnong S, T-Thienprasert J (2020) First-principles study of chromium defects in -AlO: The origin of red color in ruby. Phys Status Solidi B 257:2000159. 10.1002/pssb.202000159

[15] Abd Halim NAF, Razali WAW, Yaakob MK, Haziq Ridzwan M, Mohamed Z, Matmin J, Hashim A (2023) The role of chromium on the structural, electronic and photoluminescence properties of alumina: theoretical and experimental study. Inorg Chem Commun 149:110437. 10.1016/j.inoche.2023.110437

[16] Saito H, Suzuki K, Sato K, Kotani T (2023) Construction of model Hamiltonians for transition-metal impurities via the quasiparticle self-consistent GW method. Phys Rev B 108:035151. 10.1103/PhysRevB.108.035141

[17] Cotton FA (1990) Chemical applications of group theory, 3rd edn. Wiley, New York, pp 978–0471510949

[18] Torres AD, Moura CEV, Oliveira RR, Rocha AB (2022) Comparison among several vibronic coupling methods. J Mol Model 28:253. 10.1007/s00894-022-05230-8 35951111

[19] Rocha AB, Bielschowsky CE (2001) Intensity of the n symmetry-forbidden electronic transition in acetone by direct vibronic coupling mechanism. Chem Phys Lett 337:331–334. 10.1016/S0009-2614(01)00213-5

[20] Kresse G, Furthmuller J (1996) Efficient iterative schemes for ab initio total-energy calculations using a plane-wave basis set. Phys Rev B 54:11169–11186. 10.1103/PhysRevB.54.11169 9984901

[21] Perdew JP, Burke K, Ernzerhof M (1996) Generalized gradient approximation made simple. Phys Rev Lett 77:3865–3868. 10.1103/PhysRevLett.77.3865 10062328

[22] Blochl PE (1994) Projector augmented-wave method. Phys Rev B 50:17953–17979. 10.1103/PhysRevB.50.17953 9976227

[23] Newnham RE, Haan YM (1962) Refinement of the -AlO, TiO, VO and CrO structures. Z Kristallogr 117:235–237. 10.1524/zkri.1962.117.2-3.235

[24] Gajdos M, Hummer K, Kresse G, Furthmuller J, Bechstedt F (2006) Linear optical properties in the projector-augmented wave methodology. Phys Rev B 73:045112. 10.1103/PhysRevB.73.045112

[25] Orna MV (2013) The chemical history of color. Springer, Berlin, Heidelberg

[26] Wallin E, Andersson JM, Chirita V, Helmersson U (2004) Effects of additives in - and -alumina: an ab initio study. J Phys: Condens Matter 16:8971–8980. 10.1088/0953-8984/16/49/012

[27] Kitaoka Y, Nakamura K, Akiyama T, Ito T, Weinert M, Freeman AJ (2013) Excited Cr impurity states in AlO from constraint density functional theory. Phys Rev B 87:205206. 10.1103/PhysRevB.87.205113

[28] Miessler GL, Fischer PJ, Tarr DA (2013) Inorganic chemistry, 5th edn. Pearson, Boston

[29] Poole JCP (1964) The optical spectra and color of chromium-containing solids. J Phys Chem Solids 25:1169–1182. 10.1016/0022-3697(64)90014-9

[30] Nassau K (2001) The physics and chemistry of color: the fifteen causes of color. Wiley, New York

[31] Rocha AB, Pimentel AS, Bielschowsky CE (2002) Direct investigation of the validity of vertical approximation in the calculation of transition moment matrix elements: n transition in methyl formate. J Phys Chem A 106:181–183. 10.1016/S0009-2614(01)00213-5

[32] Zaitsev AM (2000) Vibronic spectra of impurity-related optical centers in diamond. Phys Rev B 61:12909–12922. 10.1103/PhysRevB.61.12909

[33] Heiba ZK, Mohamed MB, Wahba AM (2020) Structural, optical, mechanical, and electronic properties of Cr-doped alumina. J Mater Sci: Mater Electron 31:14645–14657. 10.1007/s10854-020-04027-w

[34] Ahmed TY, Aziz SB, Dannoun E (2024) New photocatalytic materials based on alumina with reduced band gap: a DFT approach to study the band structure and optical properties. Heliyon 10(5). 10.1016/j.heliyon.2024.e27029 PMC10926069 38468939

[35] Torres AD (2020) Calculo de intensidades de transicoes d-d nos ions complexos hexacianocobaltato(III) e tetracianoniquelato(II) por acoplamento vibronico. Trabalho de conclusao de curso (graduacao), Universidade Federal do Rio de Janeiro. https://pantheon.ufrj.br/handle/11422/11694. Accessed 12 Feb 2026

