# Supplementary material for: Ab initio simulation of the absorption spectrum of Ruby and the role of vibronic coupling
Source: J Mol Model. 2026 Oct 14;32(11):423. doi: 10.1007/s00894-026-06985-0 (PMC13668941; doi:10.1007/s00894-026-06985-0)
Supplement: Supplementary file 1 [file 894_2026_6985_MOESM1_ESM.pdf]

Suplementary information

July 21, 2026

Table S1: Percentage of energy and intensity variation for 10% distortion

**Freq 10% (cm^{-1})**	**Energy variation**	**Intensity variation**
778,36	-1.0515	-5.5605
720,62	1.1083	-0.7944
718,93	-1.1154	0.6178
718,36	-1.1154	2.2948
661,32	0.0284	-2.8244
657,88	-1.1296	-0.7061
657,64	0.0213	-0.5296
616,60	-0.0071	2.3831
549,66	0.0036	-1.2357
549,47	-0.0107	0.7944
503,80	-0.0213	-0.5296
487,31	0.0000	1.8535
486,77	-1.1403	-0.9709
430,94	0.0249	0.3530
422,50	0.0071	-0.5296
422,43	0.0142	0.0000
365,43	-0.0036	0.8826
364,81	0.0284	0.0883
288,81	-1.1332	-0.1765
269,50	-0.0071	-0.2648
268,88	-0.0107	0.0000

Table S2: Percentage of energy and intensity variation for 30% distortion

**Freq (cm^{-1})**	**Energy variation**	**Intensity variation**
778,36	-2.0887	-14.2983
720,62	3.3782	-0.9709
718,93	-3.3711	2.4713
718,36	-3.1402	4.2365
661,32	1.19	-7.3257
657,88	-3.3782	-4.5013
657,64	2.2841	-6.7079
616,60	-0.0142	8.0318
549,66	-1.1474	-4.9426
549,47	1.1367	-0.1765
503,80	1.0763	-1.1474
487,31	1.1261	1.2357
486,77	-3.4315	-3.707
430,94	2.3552	1.5887
422,50	0.0213	-1.7652
422,43	-1.1048	0.0883
365,43	1.0941	1.7652
364,81	0.0249	-0.353
288,81	-2.2663	-0.353
269,50	-0.0142	-1.0591
268,88	-1.1367	-0.2648

Table S3: Percentage of energy and intensity variation for 50% distortion

**Freq (cm^{-1})**	**Energy variation**	**Intensity variation**
778,36	-3.2361	-18.6231
720,62	5.7440	1.4122
718,93	-6.7955	8.2966
718,36	-6.7671	9.4440
661,32	3.5132	-10.2383
657,88	-7.9003	-4.7661
657,64	3.4315	-14.7396
616,60	0.0213	15.3575
549,66	-1.1580	-9.2674
549,47	2.2877	-3.2657
503,80	1.0799	-1.3239
487,31	2.2841	-2.3831
486,77	-5.7263	-4.7661
430,94	3.4528	3.3539
422,50	-0.0284	-3.3539
422,43	-1.0977	0.6178
365,43	2.2379	0.9709
364,81	-1.0905	-1.2357
288,81	-3.4386	-0.7944
269,50	-0.0071	-1.9417
268,88	-1.1403	-0.3530

Table S4: Vibrational modes, predominant atomic motions, and their effects on the calculated spectrum.

**Mode (cm^{-1})**	**Vibration type**	**Spectral effect**
778.36	Symmetric stretching	Spectral change without shoulder
720.62	Symmetric stretching	Spectral change without shoulder
718.93	Asymmetric stretching; scissoring	Strong spectral change with shoulder
718.36	Asymmetric stretching	Strong spectral change with shoulder
661.32	Scissoring	Spectral change without shoulder
657.88	Scissoring	Strong spectral change with shoulder
657.64	Scissoring	Strong spectral change with shoulder
616.60	Rocking; wagging; scissoring	Spectral change without shoulder
549.66	Asymmetric stretching; wagging	Spectral change without shoulder
549.47	Asymmetric stretching; wagging	Spectral change without shoulder
503.80	Rocking	No significant spectral change
487.31	Rocking	No significant spectral change
486.77	Scissoring	Strong spectral change with shoulder
430.94	Scissoring	Spectral change without shoulder
422.50	Wagging; rocking	No significant spectral change
422.43	Wagging; rocking	No significant spectral change
365.43	Twisting	No significant spectral change
364.81	Scissoring; twisting	No significant spectral change

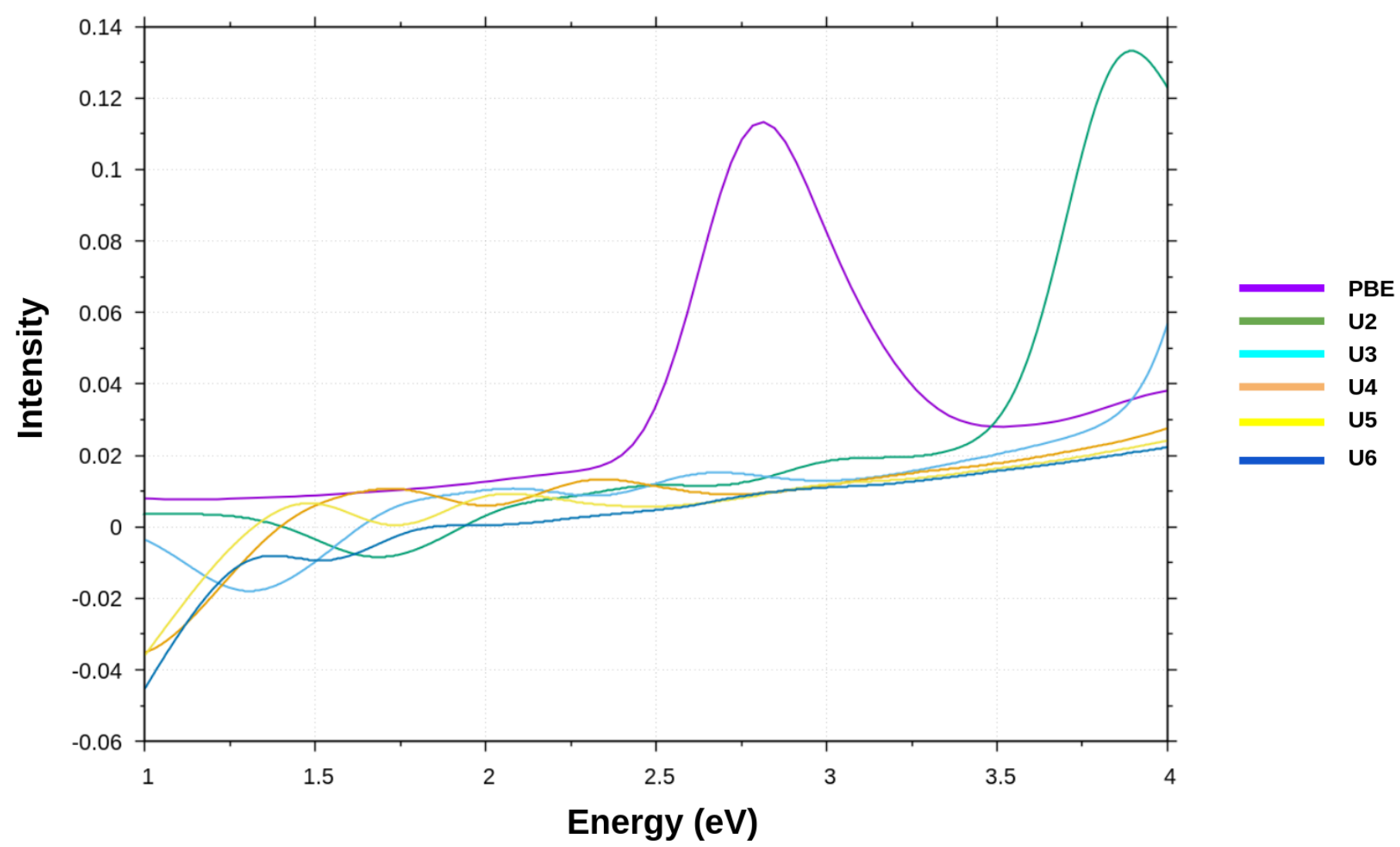

Figure S1: DFT+U calculation using different U parameters.

From Figure S1, it is possible to observe that the DFT+U calculation can reproduce the two peaks in the visible region of the spectra. On the other hand, the DFT+U calculation generate negative intensities in considerable part of the spectra. Also, the peaks have a very low intensity, when referred to PBE, which is within the method error. For U equal to 2, an intense peak it is observed at 3.8eV, but the second peak it is not observed.

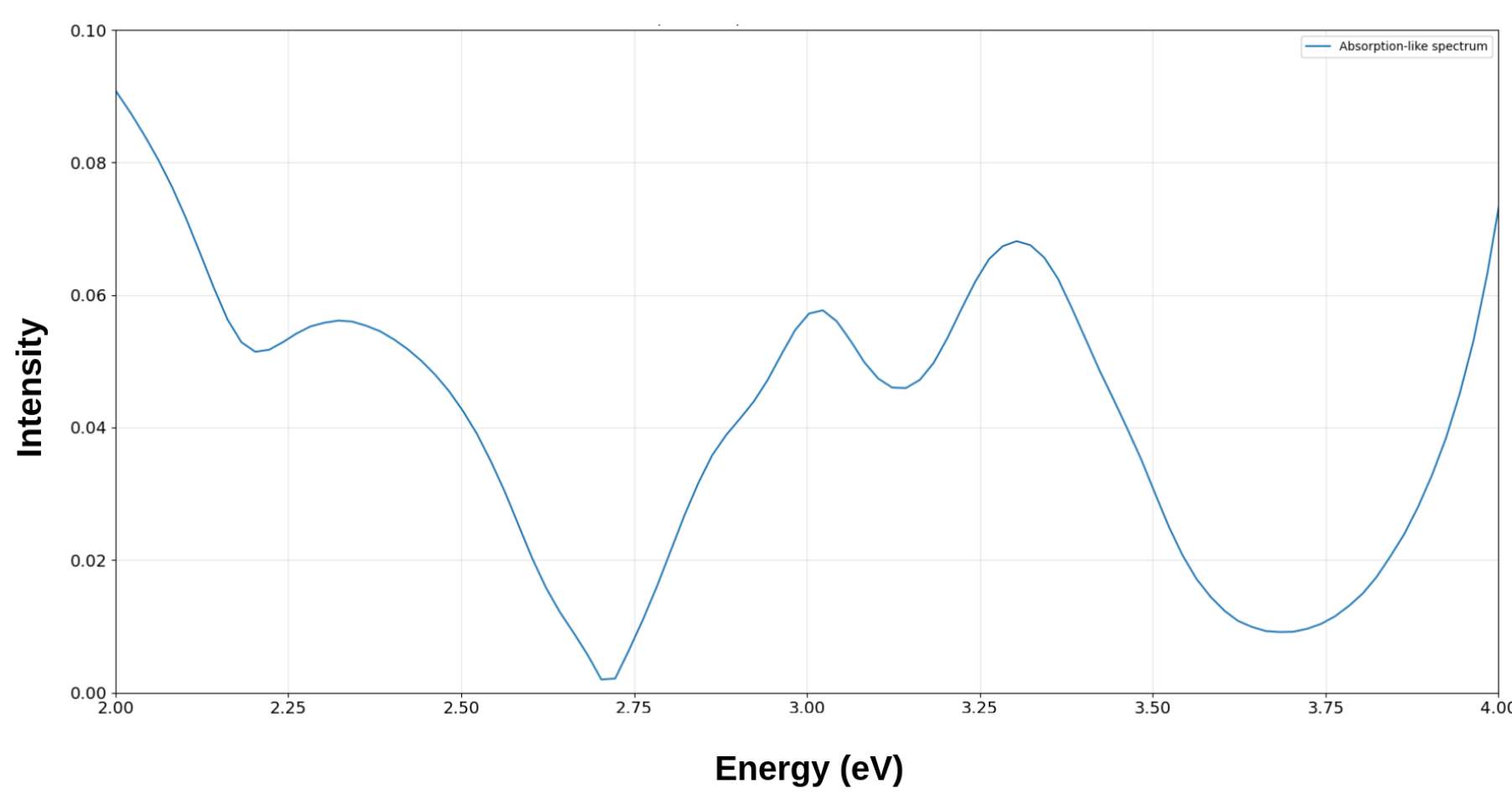

Figure S2: GW + BSE calculation for ruby in equilibrium geometry
